# The Source Parameters of Echolocation Clicks from Captive and Free-Ranging Yangtze Finless Porpoises (*Neophocaena asiaeorientalis asiaeorientalis*)

**DOI:** 10.1371/journal.pone.0129143

**Published:** 2015-06-08

**Authors:** Liang Fang, Ding Wang, Yongtao Li, Zhaolong Cheng, Matthew K. Pine, Kexiong Wang, Songhai Li

**Affiliations:** 1 The Key Laboratory of Aquatic Biodiversity and Conservation of Chinese Academy of Sciences, Institute of Hydrobiology, Chinese Academy of Sciences, Wuhan 430072, China; 2 University of Chinese Academy of Sciences, Beijing, China; 3 Sanya Key Laboratory of Marine Mammal and Marine Bioacoustics, Sanya Institute of Deep-sea Science and Engineering, Chinese Academy of Sciences, Sanya, 572000, China; Texas A&M University-Corpus Christi, UNITED STATES

## Abstract

The clicks of Yangtze finless porpoises (*Neophocaena asiaeorientalis asiaeorientalis*) from 7 individuals in the tank of Baiji aquarium, 2 individuals in a netted pen at Shishou Tian-e-zhou Reserve and 4 free-ranging individuals at Tianxingzhou were recorded using a broadband digital recording system with four element hydrophones. The peak-to-peak apparent source level (ASL__pp_) of clicks from individuals at the Baiji aquarium was 167 dB re 1 *μ*Pa with mean center frequency of 133 kHz, -3dB bandwidth of 18 kHz and -10 dB duration of 58 *μ*s. The ASL__pp_ of clicks from individuals at the Shishou Tian-e-zhou Reserve was 180 dB re 1 *μ*Pa with mean center frequency of 128 kHz, -3dB bandwidth of 20 kHz and -10 dB duration of 39 *μ*s. The ASL__pp_ of clicks from individuals at Tianxingzhou was 176 dB re 1 *μ*Pa with mean center frequency of 129 kHz, -3dB bandwidth of 15 kHz and -10 dB duration of 48 *μ*s. Differences between the source parameters of clicks among the three groups of finless porpoises suggest these animals adapt to their echolocation signals depending on their surroundings.

## Introduction

The Yangtze finless porpoise (*Neophocaena asiaeorientalis asiaeorientalis*) is a member of the *Phocoenidae* family. It has a body length between 140 and 133 cm, weighs between 40 and 70 kg and reaches sexual maturity at 4.5 and 4 years in males and females respectively [1,2]. This subspecies is critically endangered and is exclusively freshwater, endemic to the middle and lower reaches of the Yangtze River in China, as well as two connecting lakes (i.e., Poyang Lake and Dongting Lake) [3]. Similar to other odontocetes, the Yangtze finless porpoise navigates the underwater environment using high frequency echolocation clicks (> 70 kHz) [4].

The vocalizations and echolocation ability of odontocetes have been well studied in captive animals over the past half-century [5–8]. Training of captive animals has allowed researchers to successfully undertake sophisticated experiments on several odontocete species; allowing researchers to accurately characterize the animal's vocalizations under varying conditions [5]. Based on these early studies on captive animals, the use of echolocation in odontocetes for detecting targets, biosonar discrimination, recognition and classification is relatively well understood [5,8]. Recent studies on free-ranging animals have greatly improved our understanding of how odontocetes use sonar in natural environments and the diversity of echolocation signals among dolphin species as well as differences within species from different natural habitats [6,9–15] and in captivity [5,13,16,17]. The findings presented in these more recent studies also provide insights into their vocal plasticity and ability to adapt to new environments.

The vast majority of studies have focused on the vocalizations and sonar in marine odontocetes, with very few investigating the echolocation signals of freshwater odontocetes, particularly the Yangtze finless porpoise [4–7,18–20]. Previous work using a single hydrophone has shown that the Yangtze finless porpoise produces narrow-band frequency echolocation clicks of short duration (~ 100 *μ*s) with peak-frequencies at ~ 126 kHz [4], sometimes in excess of 200 dB re 1 *μ*Pa @ 1 m [20,21]. However, there is a degree of uncertainty surrounding the use of a single hydrophone in these types of experiments due to the high directionality of the sonar beam, the uncertain orientation of the phonating animal within the experimental array and the limitations in deriving accurate source levels. Minor shifts in the animal's orientation may lead to the hydrophone detecting sound pressures along the transmission axis as well as the refracted echolocation signal (termed off-axis). However, the off-axis echolocation signal will be distorted; resulting in lower received amplitudes [5,16,22,23]. Therefore, it is important to ensure that the hydrophone measures the on-axis transmission signal, rather than the off-axis signal in order to accurately characterize the signal [24]. A hydrophone array consisting of several hydrophones is a simple method for distinguishing between on- and off-axis signals.

Growing anthropogenic stresses on the Yangtze River has resulted in the finless porpoise becoming critically endangered [25–27]. As a result, there is increasing number of porpoises are being held in captivity or semi-nature environments for conservation [27]. Currently, very little is known about the adaptability of these animals to differing acoustic environments. To our knowledge, there are no published studies to directly compare the echolocation signals from captive and free-ranging Yangtze finless porpoises. Here, we present for the first time a comprehensive characterization of the echolocation signals from both captive (both in a land-based aquarium and an *in situ* holding pen) and wild Yangtze finless porpoises using a hydrophone array and high sampling rate digital recording system. The results showed differences among the temporal and spectral characteristics between captive and wild individuals. This research provides an improved understanding on the echolocation signals of the Yangtze finless porpoise and their vocal adaptability to differing environments.

## Methods

### Ethics Statement

The study was conducted under a research permit issued to the Institute of Hydrobiology of the Chinese Academy of Sciences by Fishery Management Bureau of Hubei province of China with permit number 20141105. The captive housing of all porpoises within the Baiji aquarium and Tian-e-zhou National Natural Reserve are overseen by the Institute of Hydrobiology and the Fishery Management Bureau of Hubei Province, respectively. Both organizations were consulted about the current study.

### Study Sites

**Fig 1:**
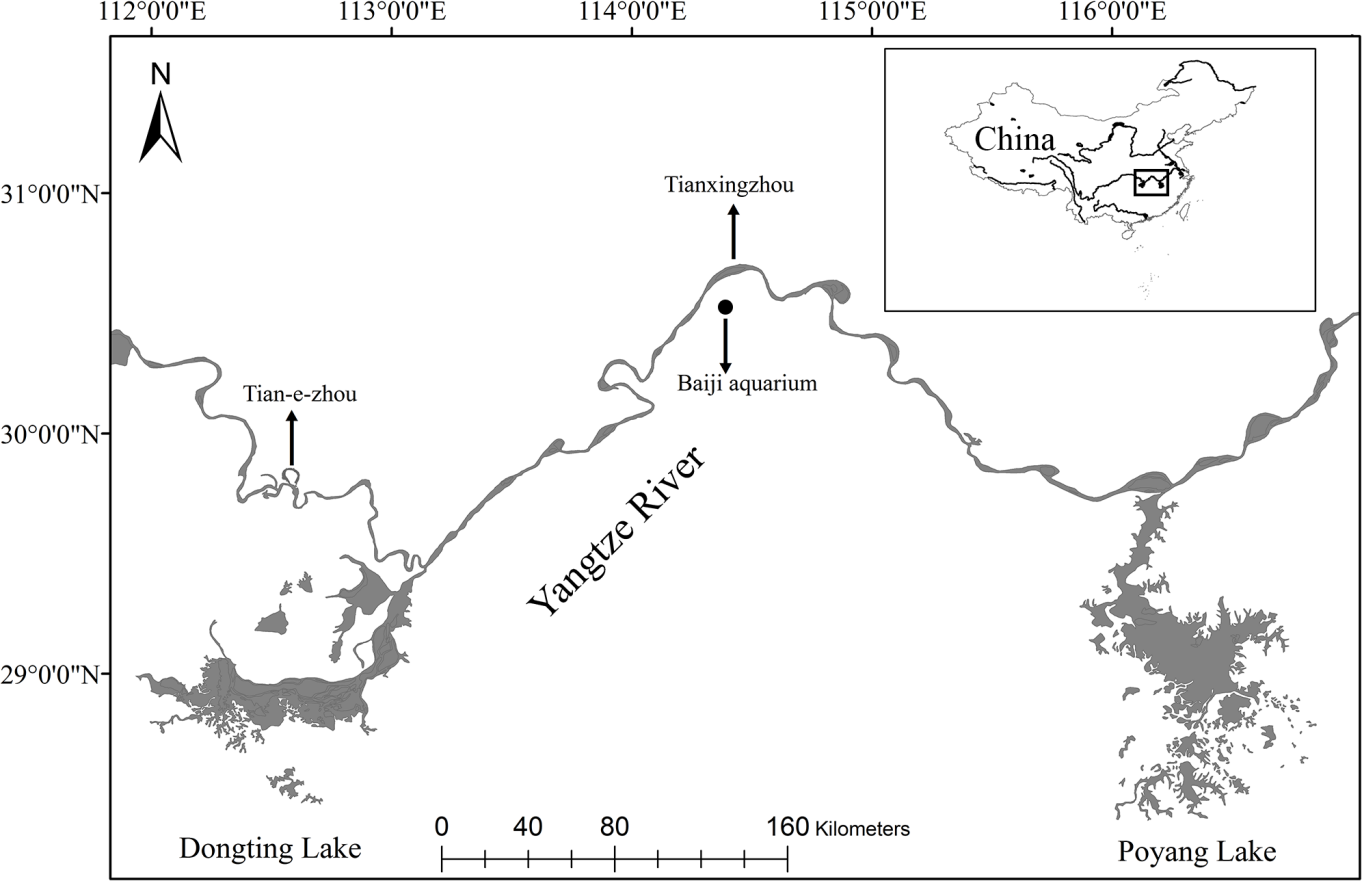
Map showing the three study sites (baiji aquarium, Tian-e-zhou National reserve and the branch of Yangtze River in Tianxingzhou).

All experiments were completed between December 2013 and January 2014 at the Institute of Hydrobiology Baiji aquarium (30° 31′ N, 114° 22′ E), the Tian-e-zhou Baiji National Natural Reserve (29° 51′ N, 112° 35′ E) and at Tianxingzhou Island (30° 41′ N, 114° 23′ E) (Fig 1). They are respectively referred to herein as the Baiji aquarium experimental group, the Shishou Tian-e-zhou Reserve experimental group and the Tianxingzhou experimental group.

At the Baiji aquarium, sounds were recorded from 7 adult finless porpoises (3 males and 4 females; 3–18 years of age; 1.36–1.47 m length; and weighed between 35 and 45 kg) within a 3 m deep tank (dimensions: 25 × 7 m). Recordings were made a short time after the last feeding of the day. At the Shishou Tian-e-zhou Baiji National Natural Reserve (an approximately 21 × 1.5 km oxbow off the Yangtze River and 20 m maximum depth), recordings of the echolocation clicks were made from 2 adult finless porpoises (a single male and female; aged approximately 8 and 6 years, respectively; 1.38–1.42 m length) within a 13 m deep pen (dimensions: 15 × 15 m with 2 cm mesh netting). At this site, recordings were made between the first and second feedings of the day. Tianxingzhou is a 3 km long island which splits the Yangtze River into two channels. During the dry season when the river level lowers, the northern channel becomes unsuitable for large cargo vessels and this area is most often occupied by small populations of finless porpoises. During this period, porpoises' are able to swim freely in an area of approximately 3000 × 150 m and a depth of 4 m. At the time when experiments were conducted, 4 porpoises (2 mother and calf pairs) were enclosed within the northern channel at Tianxingzhou.

### Recording System

**Fig 2:**
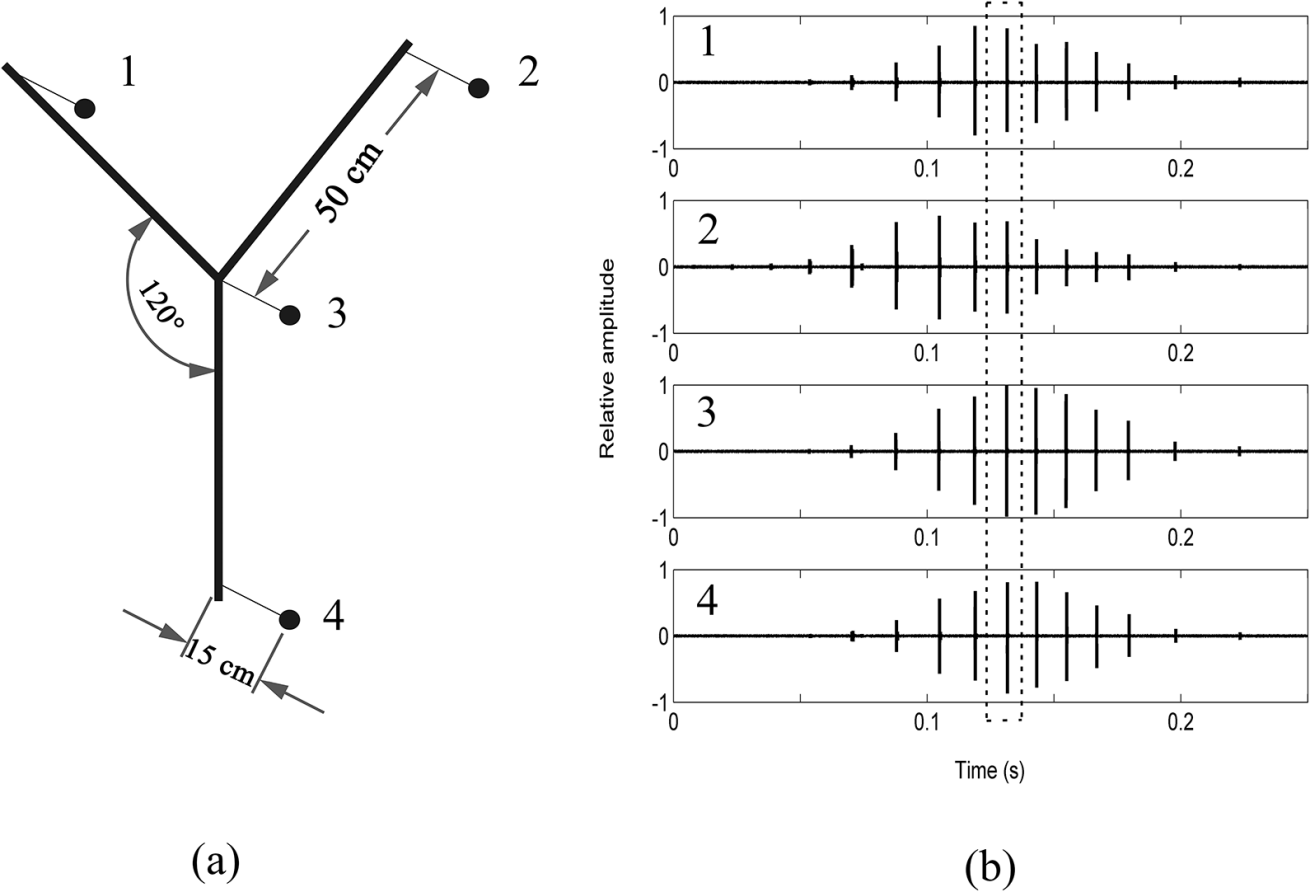
Measurement of on-axis clicks: (A) Schematic diagram of the four hydrophone symmetric star array. **The distance between the center hydrophone with the peripheral hydrophones is 50 cm, the angle of each arm is 120°and the hydrophones were attached vertically to the end of each 15 cm steel rod.** (B) A single click train received simultaneously by each hydrophone on the symmetric star array where the center hydrophone (No.3) shows the highest amplitude compared to all peripheral hydrophones (represented by the dotted box).

Echolocation clicks were recorded using a customized hydrophone array consisting of four calibrated omnidirectional hydrophones (TC-4013; 1 Hz—170 kHz flat response; -210.7 ± 3 dB re 1 V/*μ*Pa sensitivity;) connected to four VP2000 amplifiers (EC6081; 50 dB gain, bandpass filter 1–500 kHz). This type of hydrophone array has been successfully used in similar studies concerning the echolocation of odontocetes [28–32]. To control for the reverberation within the aluminum framing of the array, each hydrophone was attached to a 2 mm diameter steel rod (15 cm length) which was then welded vertically to the framing (Fig 2). Recordings data were digitalized by an cDAQ 9178 (Hi-Speed USB, 4x 32-bit counters and up to 1 MHz clock and trigger) chassis and a 4 channel NI 9223 acquisition card (± 10 V, 16-bit, 1 MS/s/ch 60 VDC, sampling rate 500 kHz). Using LabVIEW (National Instruments, USA) acoustic software, the commencing and ceasing of recordings were manually controlled.

### Data recording

The hydrophone array was deployed vertically in the water column, with the top of the array at a depth of 0.5 m from the surface and 1.5 and 1 m from the pool edge and netted pen, respectively. Recordings made at Tianxingzhou were made from the middle of the channel from a small wooden boat. At the Baiji aquarium and Tian-e-zhou Baiji National Natural Reserve, recording commenced once the hydrophone array was in place. At Tianxingzhou, recording commenced when one or more porpoises where within 100 m from the hydrophone array and continued for as long as the animal(s) where swimming towards to the hydrophone. During all experiments and recording episodes, all animals were swimming freely.

### Data analysis

Each sound recording was examined using DIAdem 2012 acoustic software (National Instruments, USA) and analyzed using customized MATLAB algorithms specifically written for these recordings. The signal-to-noise ratio (SNR) of every recorded echolocation click was calculated and only when the SNR was greater than 20 dB was that particular click considered appropriate for further analysis.

The distance between the sounds source (the porpoise) and receiver (the center hydrophone) was calculated using acoustic localization techniques based on the differences in the signal's time-of-arrival between the 4 hydrophones (see [24]). The signal's time-of-arrival was determined by cross-correlating the signal received by the array's center and peripheral hydrophones.

A series of published criteria [17,24,32,33] were used to confirm that the received echolocation signal was on-axis,thereby allowing for accurate calculation of the source parameters.

The apparent peak-to-peak source level (ASL__pp_) is defined as the peak-to-peak sound pressure level that has been back-calculated to 1 m from the sound source and is defined by:

$$ASL_{pp}=RL\;+TL$$

where *RL* is the received sound level and *TL* is transmission loss. Spherical geometric spreading of sound energy was assumed for the study and therefore, *TL* was defined by:

TL=20logR+αR

where R is the distance between the sound source and receiving center hydrophone and α is the absorption coefficient in dB m^-1^ (0.035 dB m^-1^ at 120–130 kHz and 30°C) [5,34]. The ASL_-10 dB (dB re 1 *μ*Parms) is the root mean square (RMS) sound pressure level over the 10 dB duration of the signal, and The 10 dB duration was also used to define the click time [15]. Also calculated was the energy flux density (EFD) which was the integrated sound energy over the 10 dB duration of the signal [35]. Peak and center frequencies, 3-dB bandwidth (-3 dB_BW), 10-dB bandwidth (-10 dB_BW) and RMS bandwidth (RMS_BW) were also calculated for each on-axis echolocation click [5]. The interclick interval (ICI) was also calculated and defined as the interval between the preceding on-axis click and proceeding click [5].

A descriptive statistical analysis comparing the echolocation signals collected from each of the three study sites was done using PASW Statistics 16.0 (SPSS Institute Inc., Chicago, IL, USA).

## Results

A total of 66 sound files (100 s each) containing vocalizations were recorded from the Baiji aquarium experimental group, from which 68 on-axis echolocation clicks were analyzed. A total of 68 sound files containing vocalizations where recorded from Shishou Tian-e-zhou Reserve experimental group, from which 78 echolocation clicks were analyzed. A total of 371 sound files containing vocalizations were recorded from Tianxingzhou experimental group, from which 79 clicks were analyzed.

**Fig 3:**
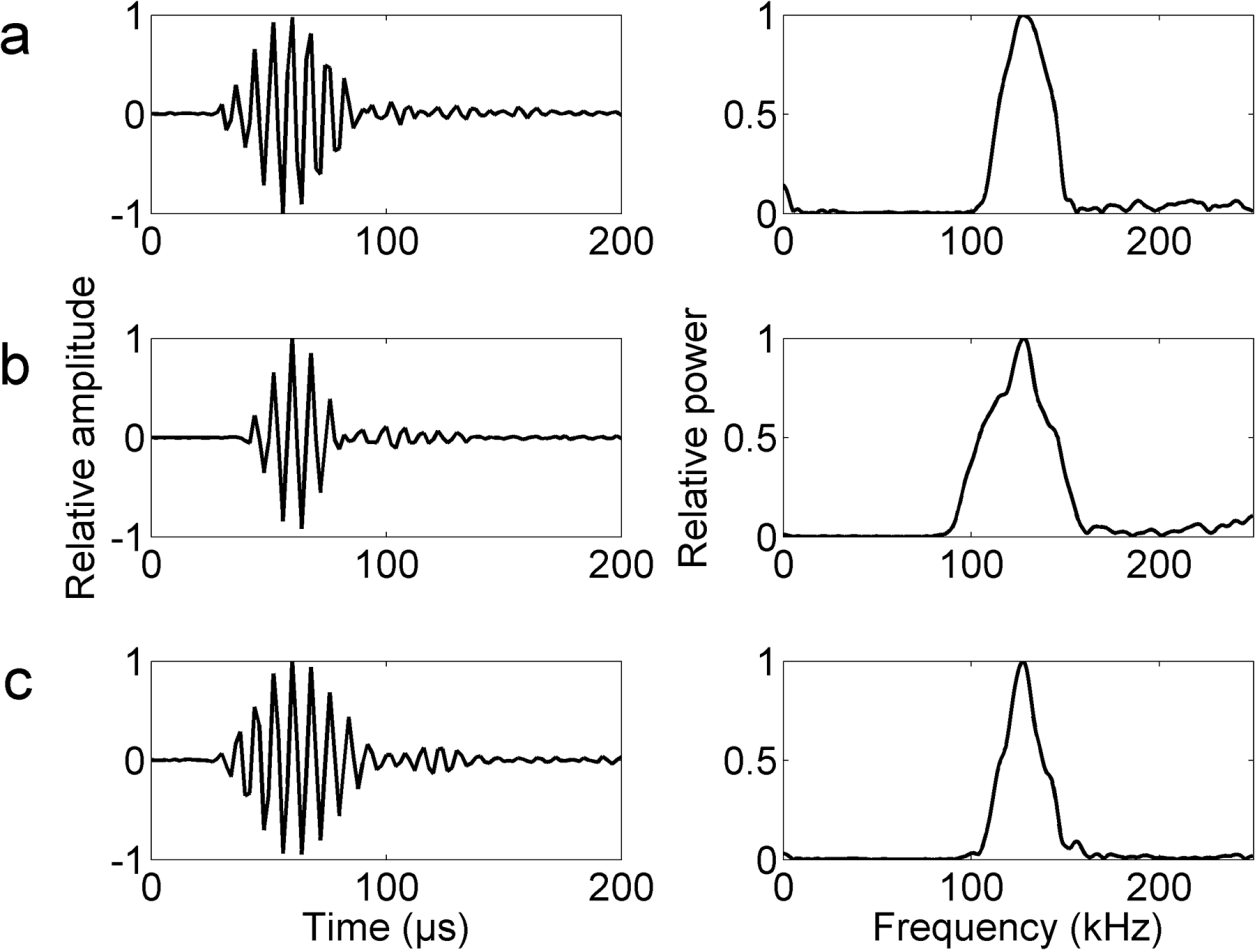
Example of a typical waveform and spectrum of clicks of finless porpoises from the three experimental groups: (A) Baiji aquarium (n = 68); (B) Shishou Tian-e-zhou Reserve (n = 78); (C) Tianxingzhou (n = 79).

Waveforms and spectral analyzes revealed similar waveforms and spectral bandwidths among the three experimental groups of porpoises (Fig 3). The average (± SD) ASL__PP_ of echolocation clicks from Baiji aquarium, Tian-e-zhou Reserve and Tianxingzhou experimental groups was 167 ± 8, 180 ± 4 and 176 ± 10 dB re 1 *μ*Pa, respectively (Table 1). The average (± SD) distances between the sound source (i.e. a porpoise) and receiving hydrophone was 4 ± 1 m, 12 ± 5 m and 29 ± 26 m within the Baiji aquarium, Shishou Tian-e-zhou Reserve and Tianxingzhou experimental groups, respectively (Table 1).

**Table 1:** Summary of the parameters of finless porpoises among the three experimental groups and other Yangtze finless porpoises measured within the mainstream of the Yangtze River.

	Present study						Previous study[18,42]
	Baiji aquarium		Tian-e-zhou Reserve		Tianxingzhou		Yangtze River
Source parameters	Average±SD	range	Average±SD	range	Average±SD	range	Average±SD
**ASL_pp (dB re 1 *μ*Pap-p)**	167±8	152–180	180 ±4	167–192	176±10	151–195	197
**ASL_-10 dB(dB re 1 *μ*Parms)**	156±7	145–171	169 ±4	158–183	165±10	144–186	n.a
**EFD_-10 dB (dB re 1 *μ*Pa** ^**2**^ **s)**	123±5	110–131	133 ±4	124–139	128±8	110–148	n.a
**-10 dB duration (μs)**	58±11	40–92	39 ±7	32–78	48±12	30–94	68±14
**Peak frequency (kHz)**	134±10	118–149	130 ±2	12–135	129±5	118–144	125±7
**Center Frequency (kHz)**	133±8	121–148	128 ±3	121–137	129±5	119–141	n.a
**-3dB_BW (kHz)**	18±6	7–37	20 ±6	12–37	22±8	10–46	20±4
**-10dB_BW (kHz)**	48±35	15–141	47 ±4	35–61	40±9	23–63	n.a
**RMS_BW (kHz)**	21±7	9–38	14 ±3	10–25	12±2	7–17	n.a
**ICI (ms)**	43±28	10–157	14 ±7	9–55	68±45	19–283	n.a
**Range from array**	4±1	1–8	12 ±5	5–19	29±26	1–86	n.a

**Fig 4:**
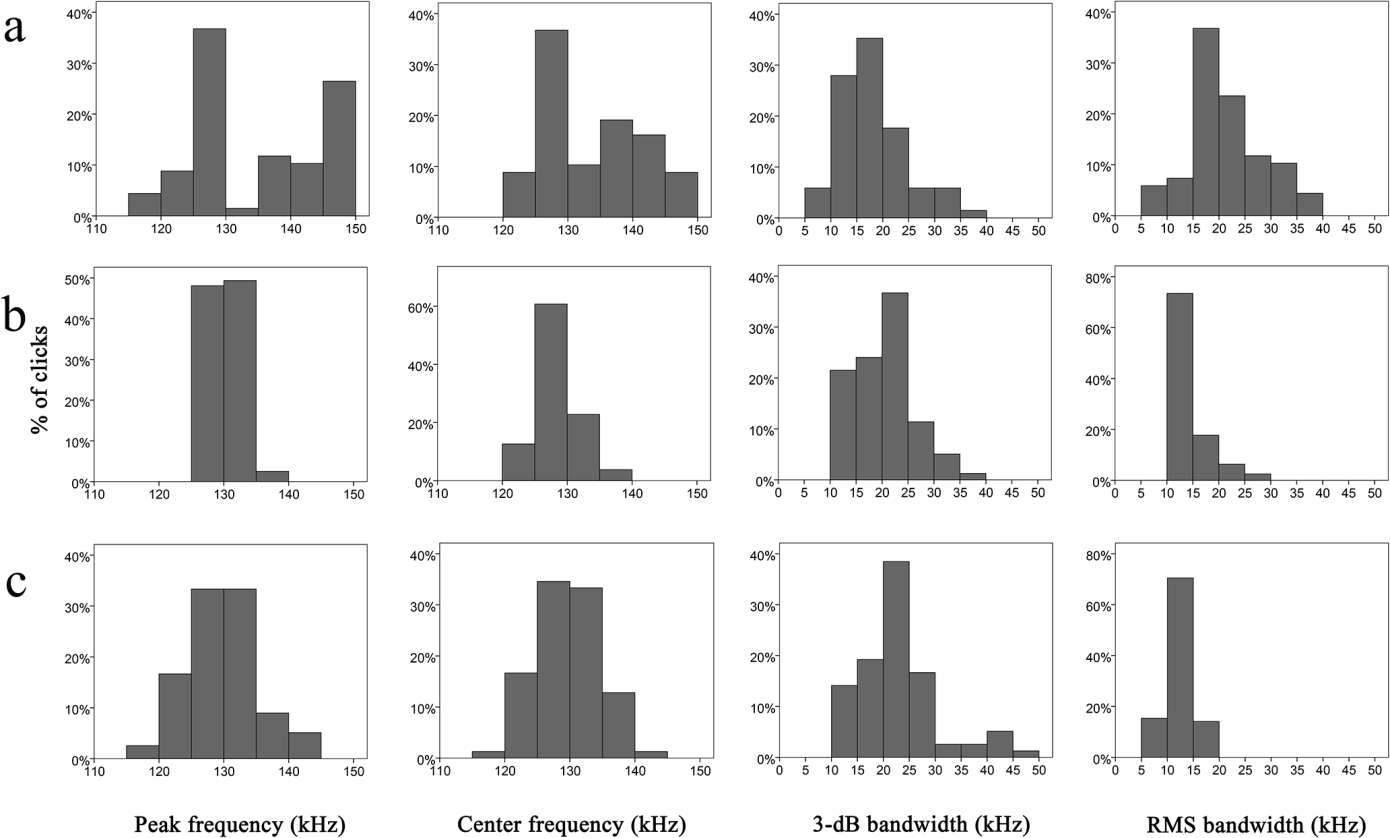
Histogram of peak frequency, center frequency, 3-dB bandwidth and RMS bandwidth of clicks of finless porpoises from the three experimental groups: (A) Baiji aquarium; (B) Shishou Tian-e-zhou Reserve; (C) Tianxingzhou.

The peak frequency of clicks recorded within the Baiji aquarium experimental group conformed to a bimodal distribution with the greatest number of clicks (36.8%) showing peak frequencies between 125 and 130 kHz and again between 145 and 150 kHz (26.5% of clicks) (Fig 4). This differed to the other two experimental groups where most clicks (97.5 and 67.0% at Shishou Tian-e-zhou Reserve and Tianxingzhou, respectively) had peak frequencies between 125 and 135 kHz (Fig 4). Similar modal distributions among center frequencies where also observed among each experimental group where the highest percentage of clicks (36.0, 68.0 and 60.8% at Baiji aquarium, Shishou Tian-e-zhou Reserve and Tianxingzhou, respectively) were between 125 and 130 kHz (Fig 4). The highest percentage of echolocation clicks occurred within the 3-dB bandwidth of 15–20 kHz (Baiji aquarium) and 20–25 kHz (Shishou Tian-e-zhou Reserve and Tianxingzhou), while the most common RMS bandwidth was within 15–20 kHz (Baiji aquarium group) and 10–15 kHz (Shishou Tian-e-zhou Reserve and Tianxingzhou experimental groups) (Fig 4).

**Fig 5:**
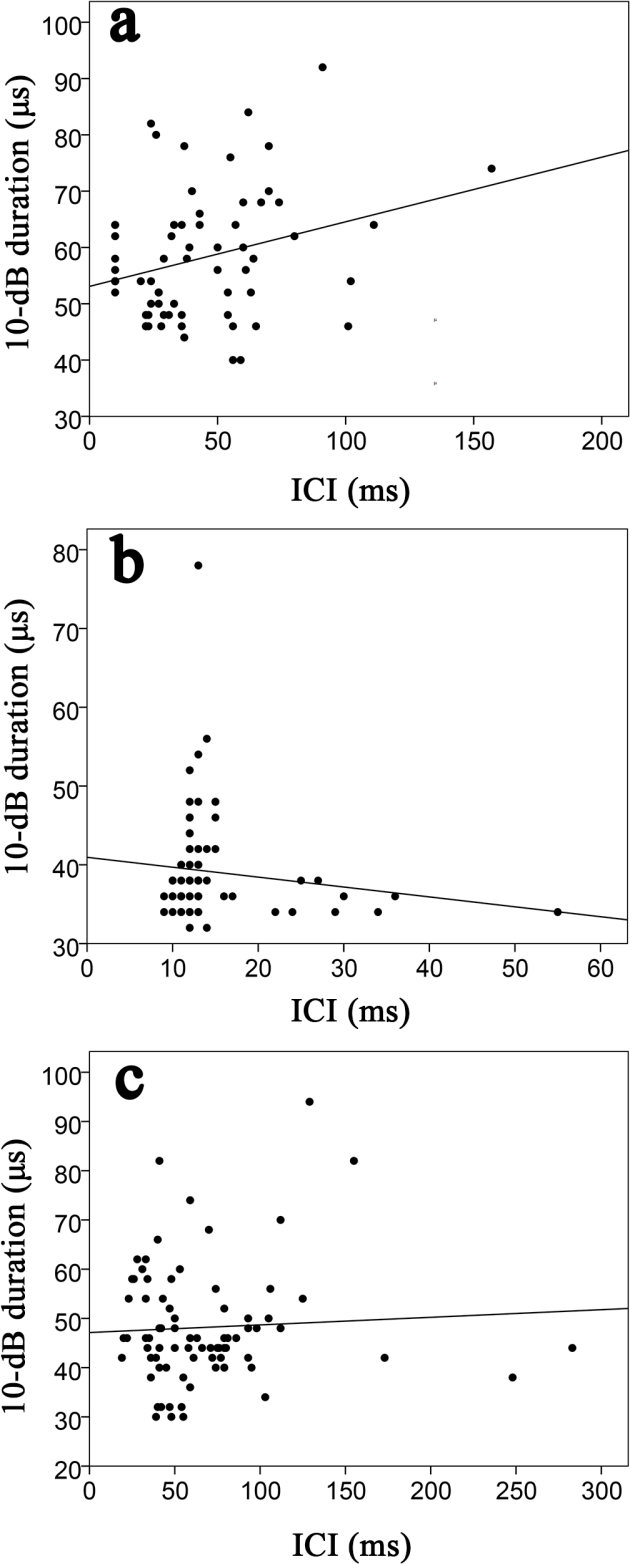
10-dB duration as a function of ICI from the three experimental groups: (A) Baiji aquarium, regression line: y = 0.11x+53.11, r^2^ = 0.08; (B) Shishou Tian-e-zhou Reserve, regression line: y = -0.13x+41.0 r^2^ = 0.02; (C) Tianxingzhou, regression line: y = 0.02x+47.11, r^2^ = 0.01.

**Fig 6:**
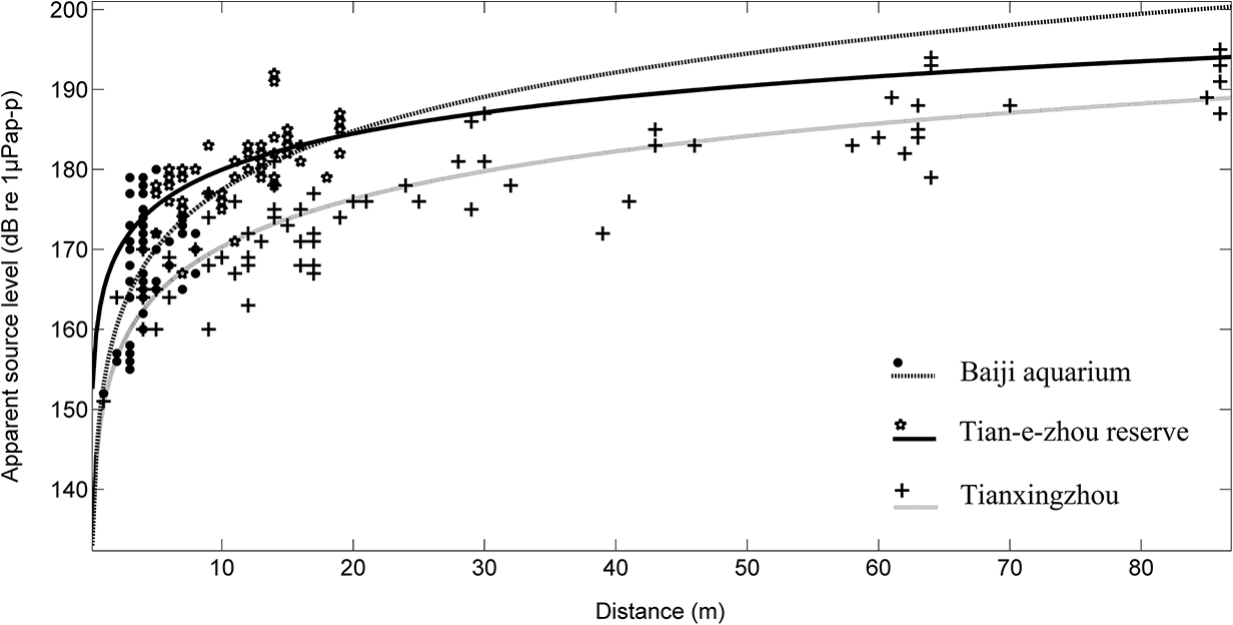
ASL as a function of the distance between individuals and the center hydrophone in the Baiji aquarium (y = 24.4log10(x)+153.06, R^2^ = 0.51), Shishou Tian-e-zhou Reserve (y = 15.0log10(x)+164.95, R^2^ = 0.47), and Tianxingzhou (y = 19.9log10(x)+150.46, R^2^ = 0.68).

**Fig 7:**
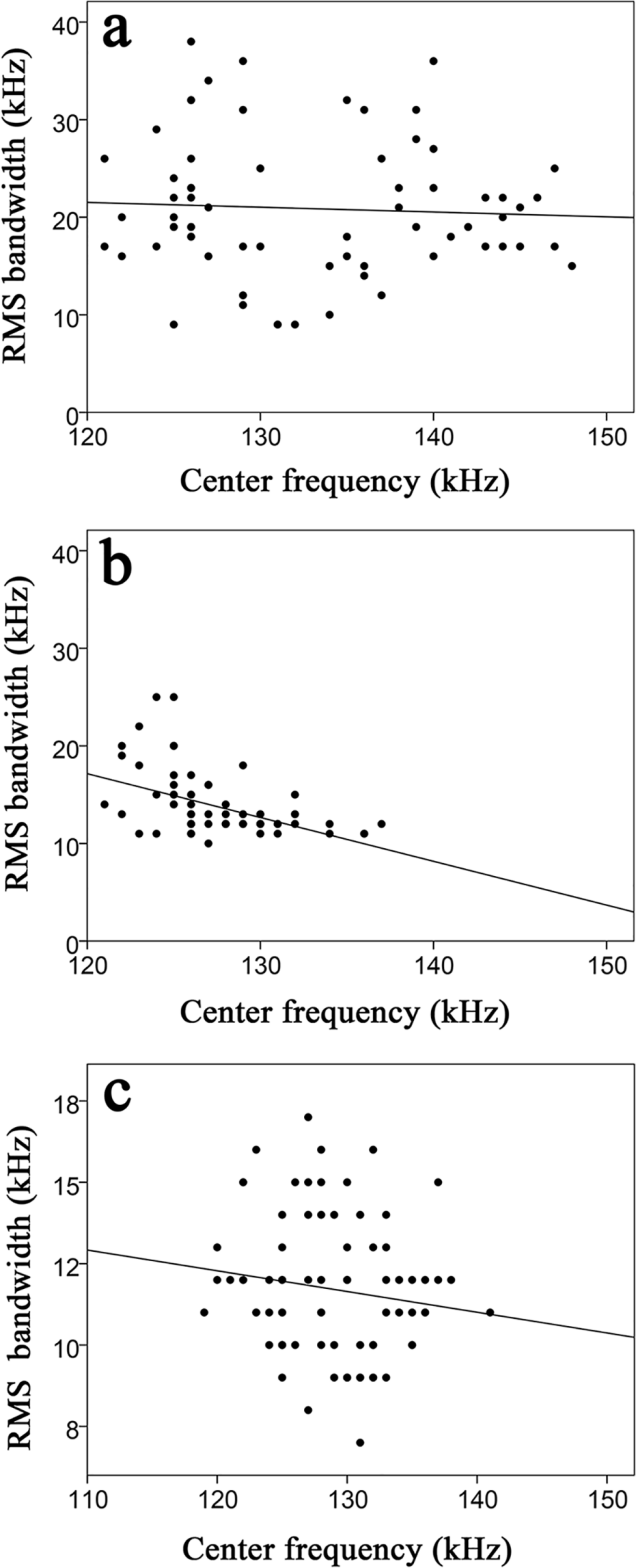
RMS bandwidth as a function of the Center frequency of finless porpoises from the three experimental groups: (A) Baiji aquarium, regression line: y = -0.01x+28.75, r^2^ = 0.01; (B) Tian-e-zhou Reserve, regression line: y = -0.44x+70.6, r^2^ = 0.22; (C) Tianxingzhou, regression line: y = -0.06x+19.34, r^2^ = 0.02.

Clicks made in quick succession tended to be shorter in duration only within the Baiji aquarium experimental group. ICIs showed an apparent positive relationship to the -10 dB duration (r^2^ = 0.08, Fig 5). Dissimilarly, the same apparent trend was not observed from the other two groups with a weak negative relationship between ICI and the -10 dB duration from within the Shishou Tian-e-zhou Reserve (r^2^ = 0.02) and Tianxingzhou (r^2^ = 0.01) experimental groups (Fig 5). Apparent source levels of clicks were found to be greater when the receiver was further away (Fig 6). There was also a weak negative relationship between the center frequencies and RMS bandwidths for all experimental groups (Baiji aquarium, r^2^ = 0.01; Shishou Tian-e-zhou, r^2^ = 0.22; and Tianxingzhou, r^2^ = 0.02) (Fig 7).

## Discussion and Conclusion

There is a critical need to better understand the echolocation behaviors of endangered species of toothed whales as anthropogenic pressure on and around the Yangtze River continues to grow. The current study presents for the first time evidence that the Yangtze finless porpoise may alter their echolocation signals in response to different environments.

The biosonar of the free-ranging Yangtze finless porpoise at Tianxingzhou showed peak frequencies between 120 and 150 kHz, ASL__pp_s' between 165 and 205 dB re 1 *μ*Pa, -10 dB durations between 50 and 175 *μ*s and -3 dB bandwidths below 30 kHz. These characteristics were quite similar with other narrow-band high frequency (NBHF) odotoncetes, such as the Harbour porpoise (*Phocoena phocoena*), Dall’s porpoise (*Phocoenoides dalli*), Hector’s dolphin (*Cephalorhychus hectorii*), Commerson dolphin (*C*. *commersonii*), Heaviside’s dolphin (*C*. *heavisidii*), hourglass dolphin (*Lagenorhynchus cruciger*), Peale’s dolphin (*L*. *australis*) and pygmy sperm whale (*Kogia breviceps*) [6,15,36–40] (Table 2). Small body sizes (~1.5 m long) between these species may explain the observed similarities in the echolocation of the finless porpoise [7].

**Table 2:** Source parameters of the echolocation of Yangtze finless porpoise (current study) and other NBHF species.

	SL_PP_	SL_EFD_	D_-10dB_	*f*_*p*_	*f*_*c*_	BW_-3dB_	BW_-10dB_	N^a^	Reference
	dB re 1 μPap-p	dB re 1 μPa^2^ s	μs	kHz	kHz	kHz	kHz		
**Heaviside’s dolphin, *C*. *heavisidii***	173	120	74	125	125	15	23	6	[39]
**Hector’s dolphin, *C*. *hectori***	177	121	57	129	128	20	30	4	[37]
**Commerson’s dolphin, *C*. *commersonii***	177	125	78	132	133	21	n/a	6	[38]
**Chilean dolphin, *C*. *eutropia***	n/a	n/a	83	126	126	18	34	1	[43]
**Hourglass dolphin, *L*. *cruciger***	197	146	115	126	128	8	13	4	[37]
**Peale’s dolphin, *L*. *australis***	185	133	92	126	129	15	n/a	6	[43]
**Harbour porpoise, *P*. *phocoena***	191	123–150	44–113	129–145	130–142	6–26	14–46	3–4	[15]
**Finless porpoise, *N.a.asiaeorientalis***	176	134	48	129	129	22	40	4	Current study

^a^Number of hydrophones.

The average ASL from the captive animals in the Baiji aquarium experimental group) was approximately 9 dB less when compared to the free-ranging animals at the Tianxingzhou field site. Individuals within the netted pen at Shishou Tian-e-zhou Reserve produced a 4 dB higher ASL than their free-ranging counterparts in Tianxingzhou. The lower intensity levels produced by the captive porpoises at Baiji aquarium may likely be due to the high-reverberation within tank environment or the short distance between the source and receiving hydrophones [5,13]. The greater sound levels produced by porpoises within the netted cage at Shishou Tian-e-zhou Reserve may be attributed to differing factors between the reserve and Tianxingzhou. However, while the animals in the netted pen inhabit a more complicated environment than at the other two sites, it is unclear whether the animals' were searching for more distant targets than at the other two study sites or whether the animals needed to produce higher sound levels (thereby increasing the echo energy to noise ratio) to detect the net [5].

While the average click ASL from the free-ranging animals in the current study (176 dB re 1 *μ*Pa) was commensurate with other NBHF species (Table 2), it did differ from a more recent study where the average ASL for free-ranging finless porpoises was 197 dB p re 1 *μ*Pa [19,20]. However, many factors could be have led to this difference. In the current study, sounds were from two calve-and mother pairs rather than a group from sole adults. There are no published studies concerning the differences in echolocation behaviors between calves and mother porpoises. Notwithstanding that, however, there is empirical data from other odontocete species which suggest that body size may directly influence the sound intensity of echolocation clicks [6,38]. It was not possible in the current study to identify if the echolocation clicks recorded by the hydrophones where from the calf or mother because the two were only observed together and the high water turbidity inhibited the use of underwater cameras. It is therefore possible that conclusions presented within this paper concerning the free-range individuals may be influenced by the quieter calf and thus possibly lowering the average ASL. Another possible reason for the differences in sound levels between the current study and Li et al. (2009) [19] could be the different environments in which either study was conducted. Li et al. (2009) [19] investigated the echolocation signals from finless porpoises within the mainstream of the Yangtze River, where maximum depths range between 30 and 50 m [19] and currents are considerably greater than at Tangxingzhou. Finally, the distance between the animals and the hydrophones could also have affected the results. In the current study, the distance between the center hydrophone and animal was always within 40 m with an average of 29 m, while in the previous study, most of the distances were greater than 50 m. It may be possible that the animals were increasing the source levels of their echolocation clicks to detect the more distant hydrophone [17,29].

Peak and center frequencies produced by the captive porpoises at the Baiji aquarium were approximately 3–4 kHz higher compared to the other two experimental groups and demonstrated a bimodal spectrum. A bimodal distribution in peak frequencies has also been described in Heaviside's dolpins [39], as well as broadband click species (such as bottlenose dolphins and beluga whales) and is thought to be related to asymmetry in the sound generator (the dorsal bursae) [41]. Related to peak frequency and playing a central role in the production of clicks, the right hand side of the dorsal bursae is approximately twice the size than the left half in broadband species, while NBHF species show less dramatic asymmetry [39]. No bimodal peak frequency distributions were observed within the other two experimental groups; thereby suggesting that the bimodal spectral distribution within the Baiji aquarium may be attributed to the high reverberation within the tank.

The ICIs of the captive finless porpoises at the Baiji aquarium showed a weak positive relationship with click duration. Odontocetes generally space their clicks apart so that the return signal from the first click is detected before the next one is emitted. Therefore the ICI is linearly correlated with target range [5]. The use of longer click duration increases the energy of the signal without increasing the amplitude (thereby conserving energy) [35]. This may be why the click duration within the current study was inversely proportional to the click ASL. A positive relationship between the ICI and click duration has also been described Heaviside's dolphin [39]. However, no relationship between ICIs and click duration were observed from the free-ranging finless porpoises at Tianxingzhou or the other captive porpoises at the Shishou Tian-e-zhou Reserve (although this may likely be due to the limited time scale over which recordings took place). Nevertheless, it does provide further evidence for a degree of plasticity in the echolocation signals of the finless porpoise.

Dolphins and porpoises can control the amplitude of the signal at the transmission phase, rather than alter the sensitivity of the receiver (termed automatic gain control (AGC)) [17,24,29,32,33]. As the porpoise approaches a target (for example, a school of fish), the echo level remains relatively constant while the emitted signal amplitude decreases with an amount corresponding to 20logR, where *R* is the distance to the target. This increases the dynamic range over which the animal may detect prey [29]. Results from the current study demonstrate the source level of echolocation signal of free-range finless porpoises decrease at a rate approximate to 20logR, similar to other echolocating odontocetes. Within the Baiji aquarium group, the source level conformed more to 24logR.

There was a negative correlation between spectra (center frequencies) and bandwidth among captive porpoises; coinciding with previous research [36]. However, while broadband species typically show a negative correlation, such as Atlantic spotted dolphins *Stenella frontalis*, Spinner dolphins *S*. *longirostris*, and Pantropical spotted dolphins *S*. *attenuata* [24,31], more research focusing on the relationship between frequencies and bandwidths in NBHF species is needed before conclusions concerning interspecies variation can be made. Nevertheless, the results from the current study suggest that the Yangtze finless porpoise alters the source parameters of their echolocation signals in response to differing environments.
